# A Procedural Faculty Development Course for Emergency Physicians Practicing in Community and Academic Settings

**DOI:** 10.5811/westjem.61860

**Published:** 2026-07-31

**Authors:** Ryan Walsh, Charles Lei, Saralyn R. Williams, Kurt Smith, Jason Lesnick, Sean Boaglio, Amanda Smith, Michael Olushoga, Jonathan Andereck, Jeremy S. Boyd

**Affiliations:** * Vanderbilt University Medical Center, Department of Emergency Medicine, Nashville, Tennessee; † Hennepin County Medical Center, Department of Emergency Medicine, Minneapolis, Minnesota

## Abstract

**Introduction:**

Maintaining competence in high-acuity, low-occurrence (HALO) procedures poses a challenge for emergency physicians as they progress past residency training or practice in lower-acuity settings. Simulation-based deliberate practice offers a feasible method to sustain procedural competence. We developed and evaluated a blended, faculty development procedural curriculum designed to improve confidence and clinical application of HALO skills across a five-hospital emergency medicine (EM) network.

**Methods:**

A faculty needs assessment and departmental quality data informed the creation of a 4-hour procedural curriculum course. The blended curriculum included asynchronous precourse procedural guides and videos followed by a 4-hour, in-person deliberate practice session led by peer EM faculty instructors. Year one procedures included cricothyroidotomy, transvenous pacing, catheter thoracostomy, neonatal resuscitation, and peritonsillar abscess drainage. Year two curriculum included fiberoptic nasotracheal intubation, pediatric airway management, ultrasound-guided pericardiocentesis, intraosseous access, lateral canthotomy, balloon tamponade of gastrointestinal hemorrhage, fascia iliaca nerve block, and orthopedic reduction/splinting. All stations used clinical equipment stocked across the enterprise. Anonymous pre- and post-course surveys measured self-reported procedural confidence and global course satisfaction. Procedure billing data and faculty self-report were used to evaluate clinical application.

**Results:**

In year one, 62 of 90 (69%) participants completed surveys which demonstrated significant increases in confidence across all procedures (*P* < .01) with high satisfaction (median 5, IQR 5–5). Year two results were similar: 67 of 87 (77%) completed surveys, again showing significant confidence gains (*P* < .01) and high satisfaction (median 5, IQR 5–5). Seventy percent (47 of 67) of year two respondents were returning participants; among them, 68% reported performing at least one procedure reviewed in year one. All reported increased confidence during clinical performance. Peritonsillar abscess drainage increased from 14 procedures precourse to 133 in year one and 83 in year two, with 35% performed in regional hospitals. We observed no change in procedure-related quality incidents.

**Conclusion:**

Our faculty procedural curriculum offers a framework that emergency departments can use to support faculty in increasing confidence in performing HALO and other low-frequency procedures. Future research should evaluate the durability of this educational approach, including how long the intervention preserves procedural confidence without decay.

## BACKGROUND

Maintaining readiness for high-acuity, low-occurrence (HALO) procedures is challenging for emergency physicians as time since residency increases and clinical exposure remains limited. A growing body of literature demonstrates that procedural skills deteriorate over time without deliberate reinforcement.^1^ In emergency medicine (EM) contexts, retention of resuscitative and invasive procedural skills decline shortly after simulation-based training despite strong initial learning.^2^–^4^

Faculty in academic environments may face greater challenges to maintaining procedural competence.^5^ Needs assessments and national surveys reveal that emergency physicians are concerned about procedural skill attrition and cite the presence of learners as a key barrier to hands-on experience.^6^ Many academic emergency departments also lack standardized systems to evaluate or maintain attending procedural skills.^7^ A recent commentary highlights the need for systems to monitor for skill decay even during residency, underscoring the need for longitudinal continuing education strategies.^8^

Simulation-based education offers a well-supported counter measure. Deliberate practice outperforms traditional clinical exposure for skill acquisition,^9^ and mastery-learning programs demonstrate durable performance gains with reductions in procedural complications.^10^,^11^ Guidance on simulation-based procedural training emphasizes structured design principles for rarely performed, high-stakes procedures, supporting its relevance for HALO preparedness.^12^

However, much of the literature focuses on trainees rather than practicing emergency physicians,^13^ targets single procedures rather than a comprehensive HALO portfolio,^14^–^16^ or emphasizes initial skill acquisition without addressing longitudinal maintenance across distributed practice environments.^17^,^18^ Few published models describe system-wide faculty development curricula and translational outcomes such as subsequent clinical application or system-level procedure use after faculty-focused curricula.^19^

Our EM faculty practice within a geographically distributed health system that includes a tertiary academic center and multiple affiliated community emergency departments. Procedural equipment, specialist availability, and opportunities for hands-on procedural experience vary across sites, creating challenges in maintaining consistent readiness for HALO procedures and contributing to procedural skill decay. These challenges highlight the need for a faculty development curriculum designed to reinforce procedural competence using the equipment and techniques employed in routine clinical practice across the system.

## OBJECTIVES

The objective of this project was to design, implement, and evaluate a longitudinal, system-wide EM faculty procedural curriculum to support readiness for HALO and other infrequently performed EM procedures across academic and community practice settings. We aimed to increase self-reported procedural confidence, promote subsequent clinical application of targeted procedures, and align training with clinically available equipment, informed by system-level quality priorities. A secondary objective was to assess faculty engagement and early implementation outcomes including participation and satisfaction.

**Population Health Research Capsule**

What do we already know about this issue?

*Procedural skills decay without reinforcement, yet many emergency medicine faculty lack standardized systems to maintain them*.

What was the research question?

*Can a faculty procedural curriculum improve procedural confidence across ED practice settings?*

What was the major finding of the study?

*Self-reported confidence improved for all procedures among participants (P < .01)*.

How does this improve population health?

*By improving faculty procedural confidence for rare, high-risk ED procedures, this curriculum may support improved care across academic and community sites*.

## CURRICULAR DESIGN

We conducted a targeted needs assessment in fall 2022 to inform curriculum content. An anonymous, voluntary survey using REDCap was completed by 78 of 105 EM faculty. We selected 23 procedures, guided by the American Board of Emergency Medicine model of clinical practice, 20 and asked faculty whether they had personally performed each within the prior three years (supervision excluded; simulation or cadaver practice included). Only 10 of 23 procedures were performed by more than 50% of respondents. These findings, together with procedures prioritized by our quality division based on complication trends, informed the initial curriculum by focusing on high-impact skills with low recent exposure. Although most selected procedures aligned with standard HALO classifications, several less acute or higher-frequency procedures were included because the needs assessment demonstrated low recent performance and they were deemed important by our faculty education and training team and departmental leadership.

Five initial index procedures were selected for a half-day course: cricothyroidotomy; transvenous pacing; percutaneous catheter thoracostomy; neonatal resuscitation; and peritonsillar abscess (PTA) drainage. We developed a blended curriculum strategy using Kern’s curriculum design framework.^21^ The curriculum combined precourse asynchronous review of our previously published and described procedural guides and publicly available instructional videos (YouTube) with a 4-hour in-person interactive session focused on deliberate practice.^22^

Two peer instructors coached each station. Instructors were selected via an internal application process and ranged from one to more than 20 years post residency (predominantly early- to mid-career). Many had fellowship training and demonstrated a track record of successful medical education teaching. Before course launch, instructors completed a 4-hour development session focused on curriculum design and simulation-based procedural training.^23^ Instructors met at least quarterly with the course directors to review postcourse survey feedback and implement any curricular refinements.

Each procedure was covered in a 45-minute session with participants rotating through the five stations. Groups were capped at four participants to prioritize coaching and hands-on practice. All stations incorporated task trainers, including primarily novel trainers as well as commercially available standard airway manikins. Ultrasound-focused stations combined standardized patients and phantom task trainers to enhance realism. Each station was stocked with the same procedure kits used across our hospitals to align training with clinical practice.

Instructor assignments were made by the course directors based on instructor preference and relevant expertise. The course was offered six times per academic year and participants were encouraged to attend one session; the curriculum was standardized across sessions. Participation was voluntary though incentivized through the department’s existing bonus structure, with attendees receiving an additional 0.75% of annual base salary. Over 90% of faculty participated.

We obtained institutional review board approval to administer anonymous surveys. Participants completed a voluntary postcourse REDCap survey; completion was not tied to any incentive. The survey was completed immediately after the course and included retrospective pre- and post-procedural confidence items and global course ratings. Confidence was measured on a 5-point Likert scale, and paired Wilcoxon signed-rank tests compared pre- and post-responses for statistical significance. Survey instruments are provided as supplementary materials (Appendix 1).

After year one, the curriculum was revised based on faculty surveys of desired procedures, input from the quality division, and review of the initial needs assessment. Year one instructor feedback and prior-year participant survey data also informed year two content; final selections were made by the course directors. The year two curriculum maintained the same five-station, 45-minute small-group format and included fiberoptic nasotracheal intubation, pediatric airway management, ultrasound-guided pericardiocentesis, intraosseous access, lateral canthotomy, balloon tamponade of gastrointestinal hemorrhage, fascia iliaca nerve block, and orthopedic reduction/splinting. Some procedures were paired within a single station to optimize hands-on time and instruction within the 45-minute session. The quality division prospectively monitored procedure-based complications via the hospital error tracking system to identify emerging trends.

## IMPACT/EFFECTIVENESS

In year one, 62 of 90 participants (69%) completed surveys. Participants demonstrated statistically significant increases in self-reported confidence across all targeted procedures (*P* < .01). Global course satisfaction was high (median 5, IQR 5–5). In year two, 67 of 87 participants (77%) completed surveys again showing confidence gains (*P* < .01) with similarly high satisfaction (median 5, IQR 5–5) (Figure). Participant demographics are shown in Table.

Among year two survey respondents, 70% (47 of 67) were returning participants. Sixty-eight percent reported performing a procedure taught in year one, and all of those respondents (100%) reported increased confidence in performing that procedure. As an example, 45% reported performing a PTA drainage. Our billing data demonstrated that in the year before implementation, emergency physicians drained 14 PTAs across our five hospitals. During year one, PTA drainages increased to 133 followed by 83 in year two. Across both years, 75 of 216 (35%) PTA drainages occurred at our four regional hospitals. These data are limited by reliance on billing records and were not consistently available for other procedures.

Evaluation of this curriculum suggests that a structured, simulation-based approach can enhance faculty confidence for HALO and other lower-frequency procedures. Program success was supported by using supplies that mirrored the clinical environment, departmental support for peer instructors, and financial incentives that encouraged broad participation. Additionally, year two respondents reported clinical application, with 68% performing procedures taught in year one. There was no change in reported quality incidents for these procedures.

Limitations of this study include reliance on self-report rather than patient-level outcomes, the absence of baseline performance or objective competency measures (limiting outcomes to confidence and selected use signals), and a 69% survey response rate with potential nonresponse bias.

## LESSONS LEARNED

Several factors were central to the program’s success. Delivering a defined curriculum over one year promoted sustained engagement and reinforced consistent objectives, maximizing cumulative impact. Aligning sessions with faculty schedules and protecting time from clinical demands reduced extraneous cognitive load from competing priorities. Small groups maximized hands-on time, a strength noted by participants. Additionally, while we partnered with outside experts for some procedures, (eg, the neonatal outreach team for our neonatal resuscitation station) the stations were led by EM faculty to maintain peer-to-peer coaching and ensure that scenarios and stations were practical and resembled our real-world clinical experiences. Finally, we encouraged faculty lunch outings after each course to enhance collegiality among faculty participants and reinforce longitudinal interest in the program.

## CONCLUSION

Our faculty procedural curriculum offers a framework that emergency departments can use to support their faculty in increasing confidence in performing HALO and other low-frequency procedures. Future research should evaluate the durability of this educational approach, including how long the intervention preserves procedural confidence without decay.

## Supplementary Information



## Figures and Tables

**Figure:**
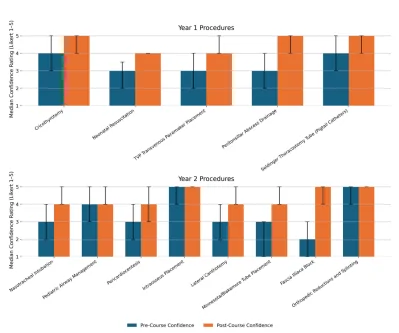
Median pre- and post-course self-reported confidence of faculty participants across procedures during the two-year intervention period. Error bars represent the interquartile range (IQR). Confidence was measured using a 5-point Likert scale (1–5). Likert scale: 1 = very uncomfortable; would consult and not perform; 2 = uncomfortable; would likely consult but might perform in select situations; 3 = comfortable with need for refresher prior to performance; 4 = comfortable; 5 = very comfortable and confident performing independently.

**Table:** Participant demographics for the 2024 and 2025 Faculty Education and Training (FacET) cohorts. One participant did not report practice environment in 2024; years since residency completion were missing for several respondents (2024: n = 20; 2025: n = 8). Hybrid faculty were defined as those who allocated a portion of their professional effort to both the academic and community practice settings.

	2024	2025	Total 2024–25
Total participants	62	67	129
Practice environment			
Academic	40 (66%)	40 (60%)	80 (63%)
Community	7 (11%)	18 (27%)	25 (19%)
Hybrid	14 (23%)	9 (13%)	23 (18%)
Years from Residency			
< 5 years	11	18	
5–10 years	11	12	
> 10 years	20	32	
