# Supplementary material for: A Procedural Faculty Development Course for Emergency Physicians Practicing in Community and Academic Settings
Source: West J Emerg Med. 2026 Jul 31;27(5):1452–6. doi: 10.5811/westjem.61860 (PMC13660566; doi:10.5811/westjem.61860)
Supplement: Supplementary file 1 [file wjem-27-1452-s001.pdf]

FacET Course Procedure Evaluation Form AY23-24

Please complete the survey below.

Thank you!

Please select your primary practice environment:	◯ Downtown Campus ◯ Community-Regional ◯ Hybrid
Practice location (select all that apply):	☐ VUH ☐ VA ☐ VWCH ☐ VBH ☐ VTH ☐ VCH
Please Identify:	◯ MD ◯ APP ◯ Other
Years out of residency	◯ 0-2 ◯ 3-4 ◯ 5-10 ◯ 11-15 ◯ >15

Cricothyrotomy

The task trainer model utilized was adequate to learn the necessary procedural skills.	Strongly disagree — Strongly agree<br>*(Place a mark on the scale above)*
Overall, the instructor was an effective educator.	Strongly disagree — Strongly agree<br>*(Place a mark on the scale above)*
The instructor explained difficult concepts clearly.	Strongly disagree — Strongly agree<br>*(Place a mark on the scale above)*
What was your comfort in performing this procedure PRIOR to this session?<br><br>Cricothyrotomy	Very uncomfortable — Very comfortable<br>*(Place a mark on the scale above)*
What is your comfort in performing this procedure AFTER this session?<br><br>Cricothyrotomy	Very uncomfortable — Very comfortable<br>*(Place a mark on the scale above)*
Overall, what were the STRENGTHS of this simulation experience?	____________________<br>(Cricothyrotomy)

Neonatal Resuscitation

The task trainer model utilized was adequate to learn the necessary procedural skills.

Strongly disagree — Strongly agree

(Place a mark on the scale above)

Overall, the instructor was an effective educator.

Strongly disagree — Strongly agree

(Place a mark on the scale above)

The instructor explained difficult concepts clearly.

Strongly disagree — Strongly agree

(Place a mark on the scale above)

What was your comfort in performing this procedure PRIOR to this session?

Neonatal Resuscitation

Very uncomfortable — Very comfortable

(Place a mark on the scale above)

What is your comfort in performing this procedure AFTER this session?

Neonatal Resuscitation

Very uncomfortable — Very comfortable

(Place a mark on the scale above)

Overall, what were the STRENGTHS of this simulation experience?

____

(Neonatal Resuscitation)

TVP Transvenous Pacemaker Placement

The task trainer model utilized was adequate to learn the necessary procedural skills.

Strongly disagree — Strongly agree

(Place a mark on the scale above)

Overall, the instructor was an effective educator.

Strongly disagree — Strongly agree

(Place a mark on the scale above)

The instructor explained difficult concepts clearly.

Strongly disagree — Strongly agree

(Place a mark on the scale above)

What was your comfort in performing this procedure PRIOR to this session?

TVP Transvenous Pacemaker Placement

Very uncomfortable — Very comfortable

(Place a mark on the scale above)

What is your comfort in performing this procedure AFTER this session?

TVP Transvenous Pacemaker Placement

Very uncomfortable — Very comfortable

(Place a mark on the scale above)

Overall, what were the STRENGTHS of this simulation experience?

____
(TVP Transvenous Pacemaker Placement)

Peritonsillar Abscess Drainage

The task trainer model utilized was adequate to learn the necessary procedural skills.

Strongly disagree — Strongly agree

(Place a mark on the scale above)

Overall, the instructor was an effective educator.

Strongly disagree — Strongly agree

(Place a mark on the scale above)

The instructor explained difficult concepts clearly.

Strongly disagree — Strongly agree

(Place a mark on the scale above)

What was your comfort in performing this procedure PRIOR to this session?

Peritonsillar Abscess Drainage

Very uncomfortable — Very comfortable

(Place a mark on the scale above)

What is your comfort in performing this procedure AFTER this session?

Peritonsillar Abscess Drainage

Very uncomfortable — Very comfortable

(Place a mark on the scale above)

Overall, what were the STRENGTHS of this simulation experience?

____
(Peritonsillar Abscess Drainage)

Seldinger Thoracostomy Tube (Pigtail Catheters)

The task trainer model utilized was adequate to learn the necessary procedural skills.

Strongly disagree — Strongly agree

(Place a mark on the scale above)

Overall, the instructor was an effective educator.

Strongly disagree — Strongly agree

(Place a mark on the scale above)

The instructor explained difficult concepts clearly.

Strongly disagree — Strongly agree

(Place a mark on the scale above)

What was your comfort in performing this procedure PRIOR to this session?

Seldinger Thoracostomy Tube (Pigtail Catheters)

Very uncomfortable — Very comfortable

(Place a mark on the scale above)

What is your comfort in performing this procedure AFTER this session?

Seldinger Thoracostomy Tube (Pigtail Catheters)

Very uncomfortable — Very comfortable

(Place a mark on the scale above)

Overall, what were the STRENGTHS of this simulation experience?

____
(Seldinger Thoracostomy Tube (Pigtail Catheters))

After reviewing the various percutaneous thoracostomy devices utilized across Vanderbilt Health Emergency Departments, which device do you most prefer?

- ◯ 1. AK-1500: Arrow Pneumothorax Kit (Catheter over needle) 8fr 16cm
- ◯ 2. AK-01601: Arrow Percutaneous Cavity Drainage Catheterization kit (Seldinger) 14fr 23cm
- ◯ 3. G56537: Cook Wayne Pigtail Catheter Kit (Seldinger)14fr 29cm
- ◯ 4. G56533: Cooke Wayne Pigtail Catheter Kit (Catheter over needle) 14fr 29cm

(Seldinger Thoracostomy Tube (Pigtail Catheters))

Ultrasound Guided IV

The task trainer model utilized was adequate to learn the necessary procedural skills.

Strongly disagree — Strongly agree

(Place a mark on the scale above)

Overall, the instructor was an effective educator.

Strongly disagree — Strongly agree

(Place a mark on the scale above)

The instructor explained difficult concepts clearly.

Strongly disagree — Strongly agree

(Place a mark on the scale above)

What was your comfort in performing this procedure PRIOR to this session?

Very uncomfortable — Very comfortable

(Place a mark on the scale above)

What is your comfort in performing this procedure AFTER this session?

Very uncomfortable — Very comfortable

(Place a mark on the scale above)

Overall, what were the STRENGTHS of this simulation experience

____
(Ultrasound Guided IV)

FacET Course Overall Feedback

Overall, I found this course to be valuable.

Strongly disagree — Strongly agree

(Place a mark on the scale above)

Overall, I felt the course managed time well and the return on the investment of my time was appropriate.

Strongly disagree — Strongly Agree

(Place a mark on the scale above)

What changes would you suggest to optimize this learning experience?

____
(Course)

What procedures would you like us to cover in the future?

____
(Course)

FacET Course Procedure Evaluation Form AY24-25

Please complete the survey below.

Thank you!

Please select your primary practice environment:	◯ Downtown Campus ◯ Community-Regional ◯ Hybrid
Practice location (select all that apply):	☐ VUH ☐ VA ☐ VWCH ☐ VBH ☐ VTH ☐ VCH
Please Identify:	◯ MD ◯ APP ◯ Other
Years out of residency	◯ 0-2 ◯ 3-4 ◯ 5-10 ◯ 11-15 ◯ >15
Did you attend last year's FacET course?	◯ Yes ◯ No
Did you perform a procedure in the ED that was reviewed during the FacET course that you might otherwise would usually have not done or deferred to a consultant? (Transvenous Pacing, Pig tail catheter placement, Peritonsillar Abscess Drainage, Cricothyroidotomy, US guided IV placement, Neonatal Resuscitation)	◯ Yes ◯ No
Which procedures reviewed did you perform since the FacET course?	☐ Transvenous Pacer Placement ☐ Pigtail/small bore chest tube placement ☐ Neonatal Resuscitation ☐ Cricothyrotomy ☐ PTA Drainage ☐ US guide IV placement
Do you feel the FacET course helped you feel more confident in performing the procedure in the Emergency Department?	◯ Yes ◯ No

Nasotracheal Intubation

The task trainer model utilized was adequate to learn the necessary procedural skills.	Strongly disagree — Strongly agree<br>*(Place a mark on the scale above)*
Overall, the instructor was an effective educator.	Strongly disagree — Strongly agree<br>*(Place a mark on the scale above)*
What was your comfort in performing this procedure PRIOR to this session?<br><br>Nasotracheal Intubation	Very uncomfortable — Very comfortable<br>*(Place a mark on the scale above)*
What is your comfort in performing this procedure AFTER this session?<br><br>Nasotracheal Intubation	Very uncomfortable — Very comfortable<br>*(Place a mark on the scale above)*

Overall, what were the STRENGTHS of this simulation experience?

____
(Nasotracheal Intubation)

Pediatric Airway Management

The task trainer model utilized was adequate to learn the necessary procedural skills.

Strongly disagree — Strongly agree

(Place a mark on the scale above)

Overall, the instructor was an effective educator.

Strongly disagree — Strongly agree

(Place a mark on the scale above)

What was your comfort in performing this procedure PRIOR to this session?

Pediatric Airway Management

Very uncomfortable — Very comfortable

(Place a mark on the scale above)

What is your comfort in performing this procedure AFTER this session?

Pediatric Airway Management

Very uncomfortable — Very comfortable

(Place a mark on the scale above)

Overall, what were the STRENGTHS of this simulation experience?

____
(Pediatric Airway Management)

Pericardiocentesis

The task trainer model utilized was adequate to learn the necessary procedural skills.

Strongly disagree — Strongly agree

(Place a mark on the scale above)

Overall, the instructor was an effective educator.

Strongly disagree — Strongly agree

(Place a mark on the scale above)

What was your comfort in performing this procedure PRIOR to this session?

Pericardiocentesis

Very uncomfortable — Very comfortable

(Place a mark on the scale above)

What is your comfort in performing this procedure AFTER this session?

Pericardiocentesis

Very uncomfortable — Very comfortable

(Place a mark on the scale above)

Overall, what were the STRENGTHS of this simulation experience?

____
(Pericardiocentesis)

Intraosseous Placement

The task trainer model utilized was adequate to learn the necessary procedural skills.

Strongly disagree — Strongly agree

(Place a mark on the scale above)

Overall, the instructor was an effective educator.

Strongly disagree — Strongly agree

(Place a mark on the scale above)

What was your comfort in performing this procedure PRIOR to this session?

Intraosseus Placement

Very uncomfortable — Very comfortable

(Place a mark on the scale above)

What is your comfort in performing this procedure AFTER this session?

Intraosseus Placement

Very uncomfortable — Very comfortable

(Place a mark on the scale above)

Overall, what were the STRENGTHS of this simulation experience?

____
(IO placement)

Lateral Canthotomy

The task trainer model utilized was adequate to learn the necessary procedural skills.

Strongly disagree — Strongly agree

(Place a mark on the scale above)

Overall, the instructor was an effective educator.

Strongly disagree — Strongly agree

(Place a mark on the scale above)

What was your comfort in performing this procedure PRIOR to this session?

Lateral Canthotomy

Very uncomfortable — Very comfortable

(Place a mark on the scale above)

What is your comfort in performing this procedure AFTER this session?

Lateral Canthotomy

Very uncomfortable — Very comfortable

(Place a mark on the scale above)

Overall, what were the STRENGTHS of this simulation experience?

____
(Lateral Canthotomy)

Minnesota/Blakemore Tube Placement

The task trainer model utilized was adequate to learn the necessary procedural skills.

Strongly disagree — Strongly agree

(Place a mark on the scale above)

Overall, the instructor was an effective educator.

Strongly disagree — Strongly agree

(Place a mark on the scale above)

What was your comfort in performing this procedure PRIOR to this session?

Minnesota/Blakemore Tube Placement

Very uncomfortable — Very comfortable

(Place a mark on the scale above)

What is your comfort in performing this procedure AFTER this session?

Minnesota/Blakemore Tube Placement

Very uncomfortable — Very comfortable

(Place a mark on the scale above)

Overall, what were the STRENGTHS of this simulation experience

____
(Minnesota/Blakemore Tube)

Fascia Illiaca Block

The task trainer model utilized was adequate to learn the necessary procedural skills.

Strongly disagree — Strongly agree

(Place a mark on the scale above)

Overall, the instructor was an effective educator.

Strongly disagree — Strongly agree

(Place a mark on the scale above)

What was your comfort in performing this procedure PRIOR to this session?

Fascia Illiaca Block

Very uncomfortable — Very comfortable

(Place a mark on the scale above)

What is your comfort in performing this procedure AFTER this session?

Fascia Illiaca Block

Very uncomfortable — Very comfortable

(Place a mark on the scale above)

Overall, what were the STRENGTHS of this simulation experience

____
(Fascia Illiaca Block)

Ortho Reductions and Splinting

The task trainer model utilized was adequate to learn the necessary procedural skills.

Strongly disagree — Strongly agree

(Place a mark on the scale above)

Overall, the instructor was an effective educator.

Strongly disagree — Strongly agree

(Place a mark on the scale above)

What was your comfort in performing this procedure PRIOR to this session?

Orthopedic Reductions and Splinting

Very uncomfortable — Very comfortable

(Place a mark on the scale above)

What is your comfort in performing this procedure AFTER this session?

Orthopedic Reductions and Splinting

Very uncomfortable — Very comfortable

(Place a mark on the scale above)

Overall, what were the STRENGTHS of this simulation experience

____
(Ortho Reductions and Splinting)

FacET Course Overall Feedback

Overall, I found this course to be valuable.

Strongly disagree — Strongly agree

(Place a mark on the scale above)

Overall, I felt the course managed time well and the return on the investment of my time was appropriate.

Strongly disagree — Strongly Agree

(Place a mark on the scale above)

What changes would you suggest to optimize this learning experience?

____
(Course)

What procedures would you like us to cover in the future?

____
(Course)

Did you review the precourse material?

◯ Yes ◯ No
